# Delivering HIV care in challenging operating environments: the MSF experience towards differentiated models of care for settings with multiple basic health care needs

**DOI:** 10.7448/IAS.20.5.21654

**Published:** 2017-07-21

**Authors:** Charles Ssonko, Lucia Gonzalez, Anita Mesic, Marcio Silveira da Fonseca, Jay Achar, Nadia Safar, Beatriz Martin, Sidney Wong, Esther C. Casas

**Affiliations:** ^a^ Medécins Sans Frontières, Operational Center Amsterdam, London, UK; ^b^ Medécins Sans Frontières, Operational Center Amsterdam, Amsterdam, The Netherlands

**Keywords:** fragile contexts, HIV, model of care, conflict, continuum of care

## Abstract

**Introduction**: Countries in the West and Central African regions struggle to offer quality HIV care at scale, despite HIV prevalence being relatively low. In these challenging operating environments, basic health care needs are multiple, systems are highly fragile and conflict disrupts health care. Médecins Sans Frontières (MSF) has been working to integrate HIV care in basic health services in such settings since 2000. We review the implementation of differentiated HIV care and treatment approaches in MSF-supported programmes in South Sudan (RoSS), Central African Republic (CAR) and Democratic Republic of Congo (DRC).

**Methods**: A descriptive analysis from CAR, DRC and RoSS programmes reviewing methodology and strategies of HIV care integration between 2010 and 2015 was performed. We describe HIV care models integrated within the provision of general health care and highlight best practices and challenges.

**Results**: Services included provision of general health care, with out-patient care (range between countries 43,343 and 287,163 consultations/year in 2015) and in-patient care (range 1076–16,595 in 2015). By the end of 2015 antiretroviral therapy (ART) initiations reached 12–255 patients/year. A total of 1101 and 1053 patients were on ART in CAR and DRC, respectively. In RoSS 186 patients were on ART when conflict recommenced late in 2013. While ART initiation and monitoring were mostly clinically driven in the early phase of the programmes, DRC implemented CD4 monitoring and progressively HIV viral load (VL) monitoring during study period. Attacks to health care facilities in CAR and RoSS disrupted service provision temporarily. Programmatic challenges include: competing health priorities influencing HIV care and need to integrate within general health services. Differentiated care approaches that support continuity of care in these programmes include simplification of medical protocols, multi-month ART prescriptions, and community strategies such as ART delivery groups, contingency plans and peer support activities.

**Conclusions**: The principles of differentiated HIV care for high-quality ART delivery can successfully be applied in challenging operating environments. However, success heavily depends on specific adaptations to each setting.

## Introduction

Unprecedented global efforts during the past 15 years have brought notable progress in access to ART services worldwide [1,2]. Seventeen million people living with HIV (PLHIV) were initiated on ART by end 2015, resulting in 35% decrease in AIDS-related deaths since 2005, with 3/4 of them still occurring in sub-Saharan Africa [2]. Building on this success, in 2015 UNAIDS launched a new global HIV strategy to prevent 75% of new infections by 2020 and shift the global needs of ART programmes by 2030 [3,4]. The World Health Organization, national governments and major donors are promoting differentiated-care approaches to achieve those goals [5–7].

While international HIV funding and key policies have mainly been focused on high-burden countries and HIV “hotspots,” progress in scaling up programmes to give care to PLHIV in context that are very unstable or highly vulnerable to conflict in the African region has been poor [6]. These settings, also referred as challenging operating environments (COE), are confronted by chronic multiple unmet basic health care needs, and fragmented and fragile health systems [8]. Often, acute conflict further exacerbates disruptions of health care provision and sudden population movements prompting health actors and international funding agencies to design strategies that address unmet health care needs [7].

MSF works in 19 countries across the world, and at the end of 2015 was supporting HIV care for more than 340,700 PLHIV and ART delivery to 260,000 people [6]. Since the early 2000s, a strategy to integrate HIV services into the basic health care model in unstable contexts was implemented in an effort to leverage the opportunities that primary health care offers to improve continuum of care in those settings [9].

Although countries such as DRC, RoSS and CAR have low national HIV prevalence rates (<5%), large population numbers result in expected large numbers of PLHIV, with regions and populations pockets with documented higher rates. Neglect of these populations has resulted in poor estimations of HIV prevalence and lack of stratified data, masking plausible high-risk groups. MSF advocates for a drastic improvement on access to care and scale up of ART coverage in adults, children and pregnant women to address excess mortality and morbidity and curb the HIV epidemic in these settings [6,9,10]. PLHIV are affected by identified barriers to access care that include: low visibility, high stigma and discrimination, inadequate models of care to guarantee services, limited support role of the civil society, low prioritization of HIV care, lack of political leadership and delays in the response to their needs in humanitarian crises [6]. We review differentiated HIV treatment and care approaches to enhance continuum of care in MSF programmes in the central African region, focusing on challenges and best practices for implementation.

## Methods

We conducted a descriptive analysis of routinely collected data used for programmatic monitoring of general health and HIV care programmes in CAR, DRC and RoSS between 2010 and 2015. We assessed feasibility of implementing differentiated HIV care approaches in these settings. Information from antenatal, maternity, outpatient, tuberculosis (TB), internal medicine, paediatric, nutrition and ART departments was routinely collected by respective staff on paper files and entered in routine data collection systems. A dedicated database for HIV for “Follow-up and care of HIV infection and AIDS” (FUCHIA) was used. HIV counselling and testing data was collected on laboratory and counselling rooms registers and transferred to standard aggregated data monitoring systems. All data was collated by an epidemiologist and analysed using Stata 13 (STATA13.0, Stata Corp, TX).

We present a crude quantitative description of the patients’ cohorts and describe and discuss models and strategies that enabled integration of HIV services in general health care.

This research fulfilled the exemption criteria set by the Médecins Sans Frontières Ethical Review Board for retrospective data analyses of routinely collected programmatic data. Anonymized secondary data was used and no intervention or patient contact was made for research purposes, therefore informed consent did not apply. We followed the countries-level policies for data sharing.

## Results

**Figure 1.**
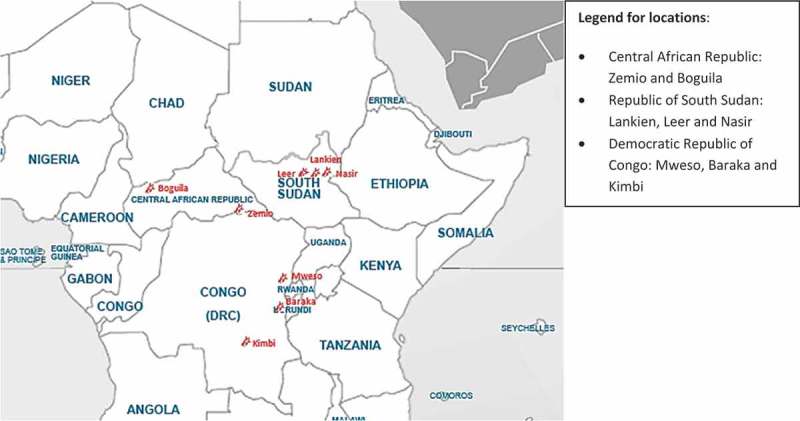
Map showing MSF-OCA projects in challenging operating environments.

In RoSS (Lankien, Leer and Nasir) MSF runs independent health care programmes supporting direct health care provision, in CAR (Zemio and Boguila) and DRC (Mweso, Baraka and Kimbi) MSF works jointly with the Ministry of Health (MoH). Health care packages include out-patient services (OPD), in-patient care (IPD), immunization programmes, surgical care in some cases, antenatal, maternity and postnatal care, care to specific main morbidities such as Kala-Azar and response to outbreaks. Figure 1 shows the geographical distribution of the MSF projects in the region.

In 2015, the number of consultations in OPD ranged between 43,343 in Zemio (CAR) and 287,163 consultations/year in Baraka (DRC), IPD activity ranged between 1076 IPD admissions/year in Zemio and 16,595 admissions/year in Baraka, and ART initiations ranged between 12 in Boguila (CAR) and 276 in Zemio in 2015. Table 1 shows a summary of services in the projects. All of them started integrating HIV care since 2010 with HIV services embedded in secondary or in upgraded primary health care.

**Table 1.** Overview MSF projects integrating HIV care in CAR, DRC and RoSS

	Central African Republic		South Sudan	Democratic Republic of Congo		
	Boguila	Zemio	Lankien	Baraka	Kimbi	Mweso
Year start HIV care	2007	2011	2011	2000	2012	2011
Local HIV prevalence	4.9 % [11]	12% [11]	2% (local ANC)	6.18% (local ANC)	2–10% (local ANC)	1.5% (local ANC)
National ART coverage (2015)	24%	24%	11%	33%	33%	33%
OPD consultations in 2015	79,278	43,343	95,065	287,163	95,809	185,051
IPD-Internal Medicine admissions 2015	N/A	1076	1363	16,595	8147	8734
Activities in the programme	OPD, HIV-TB, SGBV, community outreach activities		OPD, IPD, HIV-TB, surgery, community outreach activities	OPD, IPD, HIV-TB, DR-TB, SGBV, surgery, community outreach activities, iCCM		
Community activities	Health Promotion, defaulter tracing and sensitization for malaria, ART delivery through community-based groups and peer-support groups		Health Promotion, defaulter tracing	Health Promotion, defaulter tracing and sensitization for HIV/TB and iCCM, HIV peer-support groups		
ART initiation	Nurse	Nurse	Doctor/Clinical officer	Doctor/Nurse	Nurse	Doctor/Nurse
ART duration prescription (months)	3	3–6	3–6	1–3	1–3	1–3
Counselling activities	Lay and peer counsellors		Lay counsellors	Nurse/Lay counsellors/Mental Health officer		
CD4 access	Samples referred	Yes	Samples referred	Yes	Yes	Yes
VL access	Yes – routine monitoring		No	Yes – routine monitoring		
HIV care integrated in the basic health care package	Yes		Yes	Yes	Yes	Yes
Contingency planning	1 month ART security stock + 3–6 month run-away pack		1 month ART security stock + 3–6 month run-away pack	1 month ART security stock	1 month ART security stock + run-away pack	Not available
Activities disrupted by conflict	In 2013	Temporary stop for 2 months (2013)	No	Sporadic violence preventing patients from reaching the services timely		

ANC: antenatal care; SGBV: sexual and gender-based violence; iCCM: integrated community case management.

### Overview of HIV services in MSF projects

HIV counselling and testing services (HCT) are offered in several departments, OPD, IPD, TB department, nutrition wards, antenatal care, etc. HCT follows a general framework illustrated in MSF guidelines “Integrating HIV&TB care in basic health care package in MSF projects” prioritizing provider-initiated HCT in targeted services and high-risk patients such as those with TB, pregnant women, non-responding children in therapeutic feeding centres, patients with AIDS defining illnesses, STIs, and Kala-Azar in RoSS.

HIV and TB services are integrated in one-stop service including access to drug-resistant TB care. Clinical TB screening is done at every consultation, TB diagnosis optimized with implementation of Xpert® MTB/Rif and tracing and follow-up of contacts at health facilities.

Regarding HIV prevention, the focus is information and health promotion, condom distribution and prevention of mother to child transmission (PMTCT). PMTCT is integrated in antenatal care services as opt-out with option B+ and access to HIV DNA PCR testing for exposed babies with dry blood spot referred to external laboratories.

Table 2 shows HCT, HIV and ART initiation rates, and retention in care at 24 months after ART initiation in different settings. By the end of 2015, a total of 1101 patients were on ART in two projects in CAR, and 1063 in three DRC projects. In RoSS, 186 patients were on ART in three programmes when the conflict recommenced late in 2013. The percentage of <15 yo on ART ranged from 6.3% in CAR to 12.9% in RoSS and second line recipients ranged from 0.63% in CAR to 2.04% in DRC.

**Table 2.** Number of people tested, HIV positivity rates and ART initiations in the MSF projects in 2015

	Central African Republic^a^	South Sudan	Democratic Republic of the Congo		
	Zemio	Lankien	Baraka	Kimbi	Mweso
Number of HIV tests	3130	1851	3926	3820	6625
HIV positivity rate	9.8%	2.3%	5.2%	9.6%	2.4%
New patients enrolled in care during the period	306	20	195	233	142
Number of ART initiations	276	29	177	230	118
% ART Initiations/new HIV-positive enrolled in care over the period	90%	145%^b^	91%	99%	83%
Retention in care at 24 months after ART initiation	80.8%	N/A	54%	46%	70%

^a^Boguila (CAR) project is not represented as HTS activities were interrupted during 2015 after attack to facilities. New ART initiations came from patients already registered in pre-ART care.

^b^In Lankien, there was a carry-over of patients identified HIV-positive that had been identified earlier but could only be initiated on ART during 2015, hence a %ART initiations/new HIV-positive enrolled in care over the period is >100%.

Key programmatic HIV care and treatment approaches in these settings pivot on two strategies, HIV emergency preparedness and simplification of care.

### Emergency preparedness: Boguila (CAR) and Leer (RoSS)

When, in 2013, violent attacks to health care facilities in Boguila and Leer disrupted service provision, contingency planning for continuation of ART services had been considered and included in the programmes as part of emergency preparedness. This strategy consists of routine provision of additional ART drug supply (so-called “run-away pack”) for each patient to be used in case of unforeseen personal stock rupture. This emergency pack contains ART and cotrimoxazole supply for 3–6 months with general use instructions (see Box 1.). The quantity of drugs depends on local context and project level supply chain procedures. It is secured in a zip-lock bag for protection and confidentiality and, at every routine follow-up visit, it is inspected for expiry dates and drugs quality. Since the early programme design, a health care worker is identified as emergencies focal person in the facility and trained to manage the patient medical cards, specific recommendations and available ART stocks.

**Box 1.** Patient information topics included in the emergency “run-away pack”^a^

Basic ART information. What does contingency planning mean? Risk evaluation for interruption of ART services and compensation mechanisms for patients. What to do if ART services are interrupted: where to go, whom to contact. Drugs included in the “run-away pack,” when and how to use them.

^a^Materials for patient education are adapted to each setting and cultural environment, considering also that a proportion of patients are illiterate.

In Leer, patients fled to the swamps only with their close properties and in many cases ART was interrupted. All ART clinical files were lost; however, key information was recovered from a clinic counsellor who kept an ART registration book and traced patients effectively back to care. Until today, active severe conflict keeps services closed. MSF provided ART stocks to other facilities and supported referral of identified patients to ensure continuation of care of identified patients.

In Boguila, a total of 129 patients on ART were lost to-follow-up after the attack in 2013. A dedicated team traced back to care 58 (45%) of them either in Boguila itself or in nearby ART facilities. HCT services were interrupted and new ART initiations had to be restricted to pre-ART patients enrolled in care. The ART cohort steadily increased up to 141 patients by 2015.

### Simplification of care in CAR and DRC

Programmatic and clinical recommendations are simplified to assure quality of care in multidisciplinary teams composed by non-specialized doctors, nurses and lay providers. Job aids and checklists are used to assure standardization of care with task-shifting becoming a programmatic cornerstone. ART initiation was initially based on clinically-driven protocols without the need for additional laboratory tests. This approach assured scaled-up of care by decreasing programmatic complexity. With evolving international recommendations and technological improvements, programmes progressively incorporated access to laboratory support (CD4 monitoring) for patient follow-up, including access to VL.

#### Long-term ART retention strategies

Although the Global Fund is supporting the MoH in the ARV supply chain, the conflict in CAR has further disrupted the already weak logistics and transport capacity to deliver them to ART sites in rural areas, limiting access to ART at provincial level. Zemio has the only ART site in Haut Mbomou province. To access Zemio, patients might travel distances as far as 250 km. for every ART follow-up visit in a trip that can take three days, with worsening travelling conditions during the rainy season. The lack of public transport and high cost of alternatives to reach ART sites (trucks, motorbikes) make access unaffordable for many patients resulting in low ART intake and interruptions.

Despite that, at the end of 2015, 960 patients were on ART in Zemio, 60 (6.2%) of them under 15 yo. Twenty-four months ART retention in care was 77.3% and 81% in 2013 and 2014 respectively. To design a more patient-centred approach, support patients’ autonomy and continue improving retention in care, we incorporated wider long-term ART retention strategies in a process that continues to date, including decentralization of care, extended ART prescriptions and community-based ART delivery strategies (Box 2). Flexible long-term ART prescriptions for 3–6 months were implemented. To bring treatment closer to patient’s communities, in 2015, the project incorporated decentralization of ART to peripheral clinics and incorporated community-based ART delivery (CAGs), results are not yet available.

Community-based delivery of ART consist of the creation of ART patients groups where one group member will rotate to collect drugs refills and distribute them to the rest of the group reducing the frequency of visits to the clinic and de-linking ART delivery from clinical care.

**Box 2.** Patient eligibility criteria for enrolment into ART differentiated care strategies in Zemio

**Long ART refills**	**ART decentralization**	**Community ART groups (CAGs)**
Stable ART patient with no opportunistic infections for >6 months Good adherence for >6 months: on time visits, pill counts and no reported individual barriers taking medications 3–6 months ART stocks available One-month’s security stock (run-away pack) maintained Training of patients, pharmacy/dispensary staff and nurses	Stable ART patient for >1 year, CD4 >250 Demonstrated good adherence (% score >90) Referral to facility-care if needed Willingness to actively participate in ART support group Consent	ART Patient stable for >1 year Demonstrated good adherence (% score >90) Willingness to disclose status to CAGs members Consent
Exclusion criteria to participate in these ART models of care: Severe opportunistic infections within the last 3 months, including TB. Patients not on ART. Pregnant women.		

#### VL roll out in DRC

**Figure 2.**
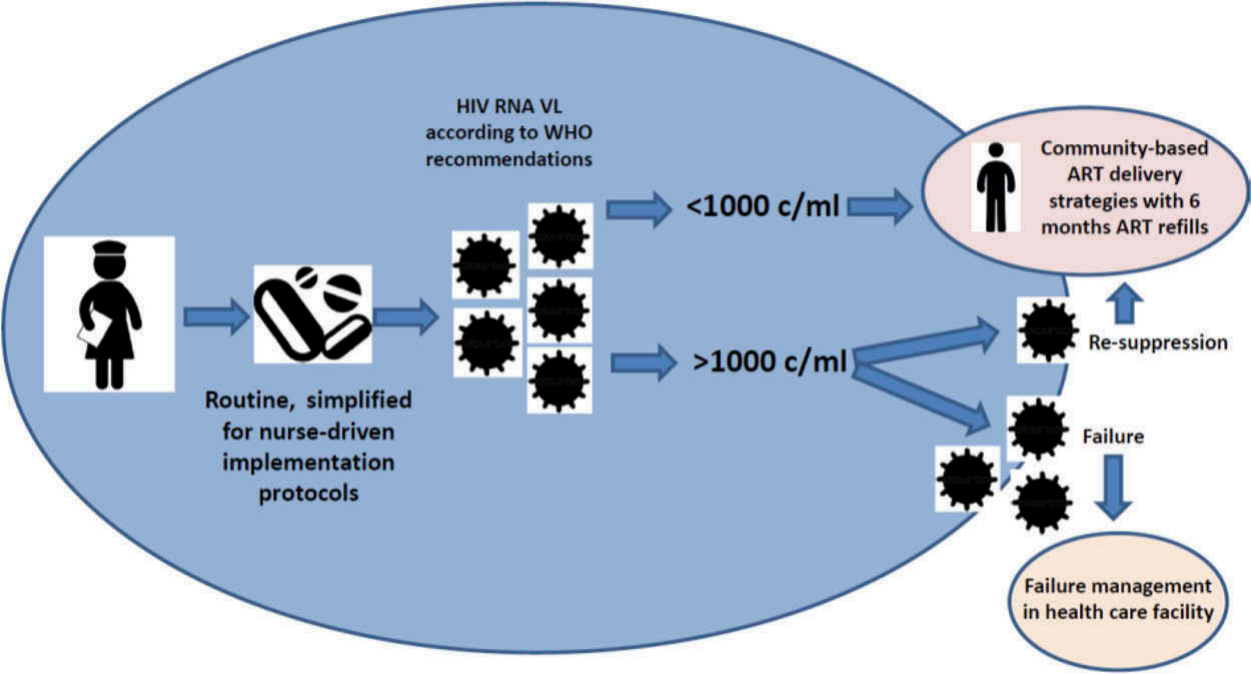
Example of use of routine VL monitoring to streamline decision-making and resource allocation in mature ART cohorts.

MSF projects in DRC progressively expanded capacity to use HIV VL using dried blood spots. From early identification of ART failure to routine treatment monitoring, the use of VL simplifies the management of growing and aging ART cohorts. Figure 2 depicts the framework of use of VL monitoring to facilitate implementation of differentiated ART service approaches and illustrates streamlined decision-making on ART delivery needs. The 1000 HIV RNA VL copies/ml threshold selects patients eligible for differentiated care models. Virologicaly suppressed patients are referred to efficient, simplified long-term ART delivery models. Non-suppressed patients trigger adherence interventions and the need to be referred for further assessment. Until 2013 in Baraka and Kimbi, VL was used to identify treatment failure, a total of 240 tests were performed in a cohort of 612 ART patients (39.2%). From 2014, routine VL monitoring was implemented and with a steady growth along time, at the end of 2015 a total of 413 samples were tested in a cohort of 988 patients (42%). In 2015, 78% and 66% of the samples in Baraka and Kimbi respectively were below the threshold of 1000 copies HIV RNA VL/ml. At the end of the same period 16/1063 patients (1.5%) were receiving second line ART.

## Discussion

Access to HIV care in high-burden settings has been the focus of international efforts over the last 15 years. In CAR, RoSS and DRC often basic lifesaving health services are not available and, despite lower HIV prevalence compared to southern African settings, the overall burden of HIV disease is high [6]. These three countries account for 94 million of people, an HIV prevalence ranging from 0.8% to 3.7% and an estimated total number of 670,000 PLHIV [12,13]. HIV is leading mortality cause amongst adults in CAR [14] and in some areas as Haut Mbomou prevalence reaches 12% [11]. ART coverage figures are at 25% in western and central African region, well below Sub-Saharan Africa (42%). It is estimated the number of people starting treatment is still outstripped by the new infections, indicating that the region is far from reaching the “tipping point” of the epidemic [15].

The GFATM defines COE as countries or regions characterized by weak governance, poor health services provision, man-made or natural crises, increased intervention of non-state actors and sharp economic declines [7,9]. MSF integrates HIV and ART care within general health care and adapts the principles of differentiated service delivery. This requires contextualized approach and a balance with the multiple competing health priorities. Despite HIV care programmes are challenging and often not considered a priority, evidence has shown that programme implementation is feasible and, although there is not yet virologic outcomes documentation, cohort outcomes are comparable to programmes in more stable settings [9,15,17]. In this review, each context highlights best practices addressing core challenges in HIV programmes: (1) deep context understanding, (2) simplification of care, (3) integration within basic health care services, (4) community involvement and (5) contingency preparation to sustain ART continuation if services are interrupted.

Boguila and Leer illustrate the relevance of contingency planning in contexts where violence, fear to violence and lack of transportation influence ART interruptions [17]. Our experience provides evidence to ensure treatment continuation and mitigate the consequences of ART interruption due to abrupt service disruption. While contingency planning ought to be context-adapted, the principle of additional provision of drugs and preparation of patients and community for the advent of sudden interruption of care is essential. In DRC and CAR, implementing long-term ART strategies and simplified care contributed to increase access and engagement in care. Simplification of protocols were centred on implementation of routine VL streamlining patient care strategies according to their needs while increasing early identification and management of treatment failure and aiming also to monitor quality of care [16]. Further evaluation of retention in care in DRC programmes and further strategies should be explored to address it.

Reduced frequency of contacts between service users and clinics for stable ART patients is a focus, and has an additional value where funding, supply chain and availability of health care workers are jeopardized. Our experience in provision of WHO recommended long-term ART refills of 3–6 months in CAR is in early stages; however, we expect stable patients decreasing the frequency of contact with clinics and de-linking clinical care from drug pick-ups.

MoH capacity widely varies posing a challenge in management of services and potential handover, particularly in settings like RoSS where the local government struggles organizing health care. Moreover, while it is beyond the scope of this paper to dive deeply into inputs and costs, these rely on MSF operational budgets including provision of national and international personnel.

The presented models of care suggest that high quality HIV interventions are feasible in such contexts. MSF takes a pragmatic approach towards barriers in the HIV continuum of care such as: addressing lack of knowledge, availing HIV testing services, increasing capacity to link to ART care and adapting systems to procedures of following a chronic health condition. An approach of this kind initially favours immediate service provision, making little attempts towards policy change or addressing upstream determinants of health. Further elaboration is needed to increase identification of new infections, tailor strategies for key populations, particularly children and adolescents, scale-up efficient use of VL and understanding the complex interplay between unstable social backgrounds and long-term retention.

Our analysis carries the limitations related to the description of operational interventions and the inherent risk of information bias related to analysis of programmatic and routine monitoring and evaluation. Also, those models do not represent a variation of standard MSF implementation but rather a feasible model to deliver care in those settings. As such, the learning is not addressed to compare outcomes of different strategies.

## Conclusions

The MSF experience delivering HIV care in COE has highlighted some key elements supporting the attainment of good outcomes. These include: integration within existing structures and services, contingency planning, and the application of principles of differentiated care including simplification, spaced ART prescriptions and community-based ART delivery.

Global efforts have demonstrated that HIV targeted investment may support fast gains in settings where the existing machinery supporting the health system can generate successful interventions. However, PLHIV are in need of urgent care in settings where health systems are weak and overloaded by competing health priorities. Contextualized differentiated service delivery approaches can support the more effective delivery of HIV services in conflict and fragile settings.
